# Identification of FadT as a Novel Quorum Quenching Enzyme for the Degradation of Diffusible Signal Factor in *Cupriavidus pinatubonensis* Strain HN-2

**DOI:** 10.3390/ijms22189862

**Published:** 2021-09-13

**Authors:** Xudan Xu, Tian Ye, Wenping Zhang, Tian Zhou, Xiaofan Zhou, Weijun Dai, Shaohua Chen

**Affiliations:** 1 Integrative Microbiology Research Centre, State Key Laboratory for Conservation and Utilization of Subtropical Agro-Bioresources, Guangdong Province Key Laboratory of Microbial Signals and Disease Control, South China Agricultural University, Guangzhou 510642, China; xuxudan@stu.scau.edu.cn (X.X.); 20182047012@stu.scau.edu.cn (T.Y.); 20191047008@stu.scau.edu.cn (W.Z.); 20171021009@stu.scau.edu.cn (T.Z.); 2 Guangdong Laboratory for Lingnan Modern Agriculture, Guangzhou 510642, China

**Keywords:** diffusible signal factor, quorum sensing, quorum quenching, *Cupriavidus pinatubonensis*, biocontrol, *Xanthomonas campestris* pv. *campestris*

## Abstract

Quorum sensing (QS) is a microbial cell–cell communication mechanism and plays an important role in bacterial infections. QS-mediated bacterial infections can be blocked through quorum quenching (QQ), which hampers signal accumulation, recognition, and communication. The pathogenicity of numerous bacteria, including *Xanthomonas campestris* pv. *campestris* (*Xcc*), is regulated by diffusible signal factor (DSF), a well-known fatty acid signaling molecule of QS. *Cupriavidus pinatubonensis* HN-2 could substantially attenuate the infection of *XCC* through QQ by degrading DSF. The QQ mechanism in strain HN-2, on the other hand, is yet to be known. To understand the molecular mechanism of QQ in strain HN-2, we used whole-genome sequencing and comparative genomics studies. We discovered that the *fadT* gene encodes acyl-CoA dehydrogenase as a novel QQ enzyme. The results of site-directed mutagenesis demonstrated the requirement of *fadT* gene for DSF degradation in strain HN-2. Purified FadT exhibited high enzymatic activity and outstanding stability over a broad pH and temperature range with maximal activity at pH 7.0 and 35 °C. No cofactors were required for FadT enzyme activity. The enzyme showed a strong ability to degrade DSF. Furthermore, the expression of *fadT* in *Xcc* results in a significant reduction in the pathogenicity in host plants, such as Chinese cabbage, radish, and pakchoi. Taken together, our results identified a novel DSF-degrading enzyme, FadT, in *C. pinatubonensis* HN-2, which suggests its potential use in the biological control of DSF-mediated pathogens.

## 1. Introduction

Black rot, caused by *Xanthomonas campestris* pv. *campestris* (*Xcc*), is a worldwide threat to plants. This disease mainly affects the aboveground parts of plants at any growth stage, resulting in a heavy loss of yield. All cruciferous vegetables, including pakchoi cabbage, broccoli, Chinese cabbage, radish, cauliflower, kale, asparagus, mustard, and turnip, are prone to suffering from black rot [1].

Quorum sensing (QS) is used by bacteria to interact with one another and coordinate their group activities. Thus, QS controls a variety of pathogen-mediated diseases in humans, animals, and plants [2]. QS systems are widely distributed in *Xcc* [3]. After infecting host plants, *Xcc* produces a series of extracellular enzymes that are important in bacterial pathogenesis. This process is linked to the signaling molecule diffusible signal factor (DSF), which was previously identified as *cis*-11-methyl-2-dodecenoic acid. Accumulated DSF results in the expression of tissue-macerating pathogenicity genes in *Xcc* [4,5]. DSF is a member of the QS signaling family, which is involved in the regulation of toxic substances produced by Gram-negative bacteria. Apart from being present in all *Xanthomonas* species, DSF is also found in *Pseudomonas aeruginosa*, *Burkholderia* sp., and marine bacteria. [6]. DSF family members’ signals are linked to bacterial toxicity, biofilm development, and pathogen antibiotic tolerance [7]. However, the main means of prevention and control of black rot are chemical pesticides and antibiotics. The excessive application of antibiotics and antimicrobials leads to drug resistance, which threatens nontarget organisms, including humans [8]. Therefore, for the consideration of human health and social security, it is becoming particularly important to find a late-model, effective, and safe prevention and control measure.

The discovery of quorum quenching (QQ) provides new avenues for the prevention and biocontrol of plant diseases. QQ disrupts the density-dependent attack of bacterial population by blocking the cell–cell communication. This novel strategy impairs the virulence of pathogens while not killing them, thus reducing the selective pressure on pathogens and delaying the evolution of QS-mediated drug resistance [9]. QQ has been engineered in plants to control pathogens and establish an active defense barrier [10]. The number of known organisms and enzymes that interfere with QS is growing, paving the way for new antimicrobial strategies to be developed [11].

In recent years, *N*-acyl homoserine lactone (AHL)-degrading bacteria and their degrading enzymes have undergone extensive studies, and their related catalytic mechanisms have been well characterized [12]. However, there is a scarcity of information about DSF-degrading bacteria and their corresponding enzymes. RpfB has been identified as an inactivation enzyme responsible for DSF degradation in *Xcc* and *Xanthomonas oryzae* pv. *oryzae* (*Xoo*) [13]. RpfB activates a variety of fatty acids into CoA esters in vitro, and furthermore, RpfB can take the place of the FadD of paradigm bacterial acyl-CoA ligase in the *Escherichia* *coli* β-oxidation pathway [14]. Thus far, the genome sequence and molecular mechanism of DSF-degrading strains are not clear, which limits the applications of these QQ strains.

In our previous study, *Cupriavidus pinatubonensis* strain HN-2, isolated from an agricultural field, exhibited superior DSF degradation activity and completely degraded 2 mM of DSF within 24 h. Moreover, strain HN-2 could reduce the black rot disease caused by *Xcc*. Application of the HN-2 strain as a biocontrol agent could substantially reduce the disease severity [15]. However, the QQ enzymes and related genes responsible for DSF degradation in strain HN-2 have not been explored. In this research, we identified a novel DSF-degrading enzyme FadT in *C*. *pinatubonensis* strain HN-2 through whole-genome sequencing (WGS) and comparative genomics. Our study further evaluated the biocontrol effects of recombinant strain *E. coli* BL21/pGEX *fadT* carrying FadT in Chinese cabbage (*Brassica pekinensis* (Lour.) Rupr.), radish (*Raphanus sativus* L.), and pakchoi (*Brassica campestris* L. ssp. *chinensis* Makino) host plants against DSF-mediated pathogens. Our findings revealed the molecular basis of highly efficient DSF-degrading *C*. *pinatubonensis* HN-2 and indicated useful genes and agents that show potential for biocontrol of black rot and other infectious diseases caused by DSF-dependent bacterial pathogens.

## 2. Materials and Methods

### 2.1. Strains, Plasmids, and Media

DSF-degrading strain *C. pinatubonensis* HN-2 was identified in our previous study [15]. Table 1 presents the strains and plasmids used. *Escherichia coli* strain DH5*α*, strain HN-2 with its derivatives, and *Xcc* strain XC1 with its derivatives were cultured in Luria–Bertani (LB) at 37, 30, and 28 °C, respectively. Antibiotics were used for cell cultures at the following concentrations: rifampicin, 50 µg/mL; ampicillin, 100 µg/mL; kanamycin, 50 µg/mL; gentamicin, 50 µg/mL; and tetracycline, 15 µg/mL. The mineral salt medium (MSM) used for the degradation of DSF in this study had the following composition: 2 g (NH_4_)_2_SO**_4_**, 0.2 g MgSO_4_·7H_2_O, 0.01 g CaCl_2_·2H_2_O, 0.001 g FeSO_4_·7H_2_O, 1.5 g Na_2_HPO_4_·12H_2_O, and 1.5 g KH_2_PO_4_ per liter of distilled water [16].

### 2.2. Whole-Genome Sequencing and Assembly

In this study, genomic data were used to gain a better understanding of the mechanisms underlying bacteria’s DSF-degrading activity. As a result, a whole-genome analysis was performed to identify the entire set of genes involved in DSF degradation. The *C. pinatubonensis* strain HN-2 was isolated from agricultural soil and deposited in Guangdong Microbial Culture Collection Center (GDMCC), China (collection number: GDMCC NO 60432) [15]. The procedure of extracting the HN-2 genome was as follows: HN-2 was first removed from the −80 °C freezer and activated on LB solid medium for two days at 20 °C. Then, the sodium dodecyl sulfate (SDS) method was used to collect the genomic DNA. Additionally, agarose gel electrophoresis was used to detect the extracted DNA, and Qubit was used to quantify it. The genome of HN-2 was sequenced using single-molecule real-time (SMRT) technology, and the sequencing was done by LC-Bio Technology Co., Ltd., Hangzhou, China. The low-quality reads were filtered using SMRT Link v5.0.1. (https://www.pacb.com/support/software-downloads/, accessed on 30 July 2020), and the reads were filtered to create a single configuration with no gaps [17,18]. The original underlying data were saved in an H5 file that included sequencing sequences, base quality values, and other details. The clean reads available for analysis were obtained by managing the quality of the original subordinate data and deleting low-quality sequences. The following information was obtained: clean reads, the total amount of data, quality value assignment, and long reads. SMRT Link V5.0.1 software was utilized to put together the reads, and Illumina short-read sequencing data were used to further polish the reads. (1) In the PacBio sequel platform, single-molecule real-time (SMRT) sequencing libraries were created with a 10 kb insert size using the SMRTbell Template kit (LC-Bio Technology Co., Ltd., Hangzhou, China), version 1.0. The whole procedure was as follows: Firstly, DNA was fragmented and concentrated. Secondly, damaged DNA ends were repaired, and the blunt ligation reaction was prepared. Then, the SMRTbell Templates were purified with 0.45XAMPure PB Beads. Moreover, the BluePippin System was used to select the size, and the DNA damage was repaired. Finally, a Qubit 2.0 Fluorometer (Thermo Scientific, Waltham, MA, USA) was used to assess the library quality, and Agilent 2100 (Agilent Technologies, Santa Clara, CA, USA) was used to detect the insert fragment size. (2) In the Illumina NovaSeq (Illumina, San Diego, CA, USA) platform, a total of 1 µg DNA from each sample was utilized as the input material for preparing the DNA sample. Sequencing libraries were created using NEBNext Ultra DNA Library Prep Kit for Illumina (NEB, Ipswich, MA, USA) according to the manufacturer’s instructions, and index codes were assigned to each sample’s sequences. In brief, sonication was used to fragment the DNA sample to 350 bp; the fragments were then A-tailed, end-polished, and ligated with a full-length adaptor for Illumina sequencing and further PCR amplification. Finally, PCR products were purified (AMPure XP system, Beckman Coulter, Beverly, CA, USA), and the size distribution of libraries was evaluated using an Agilent 2100 Bioanalyzer and quantified using real-time PCR. The wholeness of the genome assembly was evaluated using BUSCO v4.1.2 (https://busco.ezlab.org, accessed on 30 July 2020) and the Burkholderiales_odb10 benchmarking dataset.

### 2.3. Genome Component Prediction and Gene Function

The genome’s coding genes, repetitive sequences, dispersed repetitive sequences (DRs), sRNA, tRNA, rRNAs, gene islands, CRISPR, and prophages of *C.* *pinatubonensis* HN-2 were predicted. The steps were as follows: (1) GeneMarkS program (GeneMark, GA, Atlanta, USA) was used to analyze the related coding gene; (2) the RepeatMasker (Version open-4.0.5) (http://www.repeatmasker.org/, accessed on 30 July 2020) was used to predict the dispersed repetitive sequences (DRs), and Tandem Repeats Finder (TRF) Version 4.07b was used for analyzing tandem repeat sequences (TRs) [19,20]; (3) noncoding RNAs analysis, for example, transfer RNA (tRNA) gene prediction analyses, were conducted based on tRNAscan-SE analysis (Version 1.3.1), identification of ribosome RNA (rRNA) genes were done with rRNAmmer (Version 1.2) [21,22], and prediction of minute nuclear RNAs (snRNA) was done with the BLAST against the Rfam database [23,24]; (4) genomic islands were predicted with the IslandPath-DIOMB (Version 0.2) program (https://github.com/brinkmanlab/islandpath, accessed on 30 July 2020), transposons were predicted with transposon PSI using the homologous blast method [25,26], PHAST (Version 2.3) was used for predicting prophage (http://phast.wishartlab.com/, accessed on 30 July 2020), and the CRISPR Finder (Version 1.0) was used to identify clustered regularly interspaced short palindromic repeat sequences (CRISPR) [27,28].

The gene functions of HN-2 were predicted using the following databases: the Kyoto Encyclopedia of Genes and Genomes (KEGG) [29,30], Gene Ontology (GO) [31], Clusters of Orthologous Groups (COG) [32], Protein Families (Pfam), Swiss-Prot [33], the Transporter Classification Database (TCDB) [34], and the Non-Redundant Protein Database (NR) [35]. The above databases were subjected to a whole-genome BLAST search (E-value less than 1 × 10^−5^ minimal alignment length >40%), and the genes were annotated by comparing them to the genes in the above-mentioned databases. The Carbohydrate-Active Enzymes (CAZy) database was used to predict the carbohydrate-active enzymes [36].

Secretory proteins and type I–VII proteins released by pathogenic bacteria were predicted using SignalP [37]. Additionally, antiSMASH (version 2.0.2) was used to analyze the secondary metabolism gene clusters (http://antismash.secondarymetabolites.org, accessed on 30 July 2020) [38]. Furthermore, the Pathogen Host Interactions (PHI) database, the Antibiotic Resistance Genes Database (ARDB) [39], and the Virulence Factors of Pathogenic Bacteria (VFDB) database [40] were used for the analysis of drug resistance and pathogenicity [41]. Furthermore, the circular genome data visualization was graphed based on Circos software [42].

### 2.4. Genome-Based Taxonomic Classification Analysis

For a whole-genome-based taxonomic analysis, the genome sequence of *Cupriavidus pinatubonensis* HN-2 was analyzed by GTDB-Tk version 1.5.1 (database version release-202) [43], which is a toolkit used to classify prokaryotic genomes with the Genome Taxonomy Database [44,45,46,47,48,49,50]. The phylogenetic relationship between HN-2 and other bacteria was investigated by placing HN-2 in a bacterial reference tree based on 120 preselected marker genes.

### 2.5. Construction of an In-Frame Deletion Mutant and Complementation

To produce deletion mutants, *C. pinatubonensis* HN-2 wild-type was utilized as a parental strain. A 500 bp upstream and downstream fragments of the *fadT* gene were amplified by PCR and cloned into a *Hind*III-*Bam*HI-linearized pK18mobsacB vector. Triparental mating with the helper strain HB101 (RK2013) on an LB plate at 30 °C for 10 h mobilized the recombinant plasmid into strain HN-2. Cells were selected with LB medium supplemented with gentamicin and chloramphenicol. Colonies were further selected on LB plates containing 10% sucrose. ∆*fadT* mutant was verified by PCR and DNA sequencing. Further, to investigate the inhibitory effect on DSF-mediated *Xcc* pathogenicity of the *fadT* in-frame ∆*fadT* deletion mutants, biocontrol efficacy against black rot in Chinese radish (*Raphanus sativus* L.) was performed. The bacterial suspensions were inoculated on radish root slices containing suspensions of black rot pathogen *Xcc* (6 × 10^8^ CFU·mL^−1^), suspensions of *Xcc* mixed with HN-2 wild-type (6 × 10^8^ CFU·mL^−1^), and suspensions of *Xcc* mixed with *fadT* in-frame ∆*fadT* deletion mutant (6 × 10^8^ CFU·mL^−1^). Radish root slices inoculated only with distilled water were used as the control. Three replicates of each treatment were set, and the experiments were carried out at least thrice. The radish root slices were incubated at 30 °C for 2 days in an incubator. Daily records of symptoms were taken after bacterial inoculation. The macerated area was measured to evaluate the disease severity [15,51].

For complementation of ∆*fadT*, the coding sequence of *fadT* and its indigenous promoter sequence were amplified by PCR and cloned into *Hind*HIII/*Bam*HI sites of pBBR1MCS-5, generating plasmid pBBR1MCS-5-*fadT*. pBBR1MCS5-*fadT* was mobilized into ∆*fadT* mutant by triparental conjugal mating as above. The transformants were selected on LB medium containing gentamicin and chloramphenicol. Correct transformants were confirmed by PCR and DNA sequencing. The primers used for cloning and detection are all listed in Table 2.

### 2.6. Purification of FadT

Enzyme purification was performed according to the methods described in previous reports [52,53] with modifications. Cell cultures (1 L) were separated via centrifugation at 8000× *g* for 10 min. The cell pellets were resuspended in 40 mL binding buffer (140 mM NaCl, 2.7 mM KCl, 10 mM Na_2_HPO_4_, 1.8 mM KH_2_PO_4_, pH 7.0) and subjected to 400 pulses of sonication (400 W, 3 s each with a 5 s interval) in an ice-water bath [54]. Following centrifugation (13,000× *g*) at 4 °C for 30 min, the supernatant was passed through a 0.22 μm filter and applied to a GSTrap (GE Healthcare, Marlborough, MA, USA) FF column of the liquid chromatography system, AKTAPure (GE Healthcare, Marlborough, MA, USA), and GST fusion proteins were eluted via a GSTrap FF column using 10 mM reduced glutathione according to standard affinity chromatography. GST fusion proteins were digested using PreScission protease (GE Healthcare, Marlborough, MA, USA) according to the manufacturer’s instructions.

### 2.7. Enzyme Activity Assay

FadT enzyme activity was investigated using a 0.5 mL reaction mixture containing 2 mM DSF, 100 mM sodium phosphate buffer (pH 7.0), and an appropriately diluted FadT preparation. After 30 min of incubation at 35 °C, the reaction was stopped by adding 100 μL of 1 M trichloroacetic acid [55]. Concentrations of DSF residues in the reaction mixture were extracted using ethyl acetate and detected via high-performance liquid chromatography (HPLC) (Waters 2690, Milford, MA, USA) according to the method described by Ye et al. [15]. Each treatment was performed in triplicate with inactivated enzyme as control. One unit of enzyme activity (U) was defined as the amount required to catalyze the formation of 1 μmol of product per minute [52,53].

### 2.8. Virulence Tests

The virulence of the *Xanthomonas campestris* pv. *campestris* strain on host plants was tested by the leaf-clipping method as previously described [56,57]. Healthy Chinese cabbage (*Brassica pekinensis* (Lour.) Rupr.), radish (*Raphanus sativus* L.), and pakchoi (*Brassica campestris* L. ssp. *chinensis* Makino) seeds were cultivated and acclimated in sterile soil under irregular irrigation for 30 days with no application of pesticides. These plants grew in flower pots in corridors and under a ceiling to block some of the rain. The day/night temperature, humidity, and photoperiod were all set to match the natural environment during the experiment. The experimental design included the following five treatments: (1) distilled water, which was used as the control; (2) *Xcc* at 6 × 10^8^ CFU·mL^−1^; (3) *Xcc* and ∆*fadT* at 6 × 10^8^ CFU·mL^−1^; (4) *Xcc* and ∆*fadT* (*fadT*) at 6 × 10^8^ CFU·mL^−1^; (5) *Xcc* (*fadT*) at 6 × 10^8^ CFU·mL^−1^. Three replicates of each treatment were used, and the experiments were carried out at least thrice. Daily records of symptoms were taken after bacterial inoculation [51,58]. The details of the tests are as follows: The strains used in this part were already cultured in 5 mL LB medium and shaken at 30 °C and 200 rpm for 12–16 h. Then, 100 μL of bacterial suspensions were inoculated on each plant. Next, Chinese cabbage and radish were harvested after 8 days, and pakchoi was harvested after 10 days. The macerated tissue was measured to evaluate the disease severity. The percentage of maceration was calculated by comparison with the preinoculation tissue [15,51].

### 2.9. GenBank Accession Number

The 16S rDNA gene sequences of *C. pinatubonensis* HN-2 were deposited into the NCBI nucleotide sequence database under the accession number MG561941.1. The whole-genome sequence of HN-2 and sequence of *fadT* were also deposited in the NCBI database with accession numbers CP080766-CP080769 and MZ734614, respectively. The genome annotation and all annotated protein sequences of HN-2 were deposited in Figshare (https://figshare.com/s/eee14ba9310748460cf8, accessed on 22 August 2021).

## 3. Results

### 3.1. Sequencing and Analysis of the C. pinatubonensis HN-2 Genome

**Figure 1:**
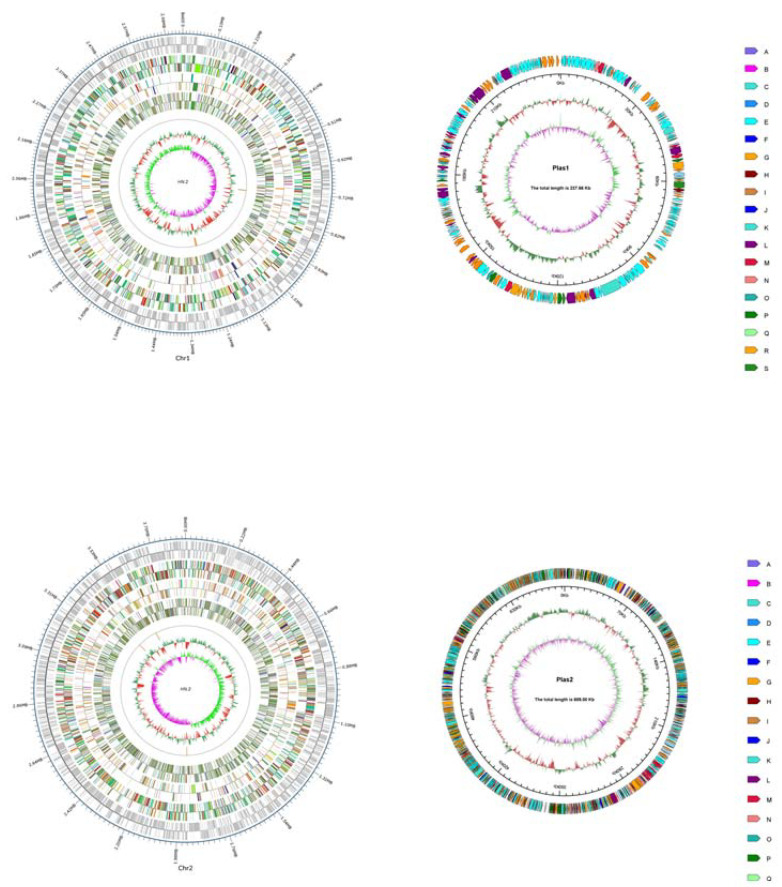
Two chromosome and two plasmid diagrams of *Cupriavidus* pinatubonensis HN-2 (red: < mean; green: > mean; the higher the peak, the greater the difference from the mean); GC skew (GC skew = (G − C)/(G + C); inward pink: G > C, outward light green: G < C) could be fully demonstrated.

The whole genome of *C. pinatubonensis* HN-2 was sequenced by both Illumina short-read and Oxford Nanopore long-read sequencing technologies, generating 4.29 and 1.63 Gb of sequencing data, respectively. A hybrid de novo assembly using both types of sequencing data resulted in four circular replicons with a total size of 7.55 Mb and a GC-content of 64.29%. The *C. pinatubonensis* HN-2 genome assembly consists of two chromosomes (Chr1: 3.89 Mb; Chr2: 2.38 Mb) and two plasmids (Plas1: 689.0 kb; Plas2: 237.7 kb) (Figure 1). BUSCO assessment of the *C. pinatubonensis* HN-2 genome assembly showed that all the 688 Burkholderiales-wide single-copy orthologs were detected as complete genes, indicating that its quality was superior.

In total, 7101 protein-coding genes, 65 tRNA genes, 18 rRNA genes, and 1 small RNA gene were predicted in HN-2. The protein-coding genes have a total length of 6,556,812 bp, accounting for 86.86% of the genome size, and an average length of 923 bp (Table S1). Table S2 contains the findings of the functional annotation of protein-coding genes and the prediction of additional genomic characteristics. There were 14 gene islands with total and average lengths of 183,507 and 13,108 bp, respectively, and the statistical map of gene distribution in the gene island is shown in Figure S1. There were also four preprophages with total and average lengths of 269,803 and 67,450.8 bp, respectively.

There were 6747, 655, 5451, 4681, 2669, and 589 coding genes annotated by NR, KEGG, COG, GO, SwissProt, TCDB, PHI, and CAZy. From the GO database, 2544 complete CDSs (coding sequences) were obtained from the coding region prediction of the strain HN-2 genome, accounting for 89.23% of the total genome. There were 2512 genes involved in the metabolic process, representing the largest number of genes, followed by 2326 and 2247 genes involved in the cellular process and catalytic activity, respectively. Figure S2 presents the classification of HN-2 functional genes in the GO database. The COG database functionally classifies 3299 CDSs, accounting for 88.99% of the total number of coding genes, and Figure S3 presents the classification of HN-2 functional genes in the COG database. There were a total of 671 genes belonging to general predicted (R) functions that were not explicitly labeled to a specific group among these functional genes. Next, there were 623, 599, 513, and 499 genes with the functions of energy production and conversion (C), transcription (K), amino acid transport and metabolism (E), and lipid transport and metabolism (I), respectively. The above two databases provide a clearer view of the relevant functional properties of strain HN-2, in which genes with functions in metabolism and catalytic activity are relatively abundant, providing references for subsequent studies on genes that may be closely related to the function of strain HN-2 in DSF degradation. Based on the KEGG database, 223 genes were predicted as xenobiotic biodegradation and metabolism genes. According to the comparison of the database, the quantity and species of biodegradation metabolism pathways were abundant in the genome of strain HN-2, and this result was mutually confirmed with the results annotated by the GO and COG databases. Other KEGG pathway annotations are enumerated in Figure S4. The predicted genes were also annotated against the CAZy database, and the classification annotation results, statistical tables, and individual statistics plots are displayed in Figure S5.

**Figure 2:**
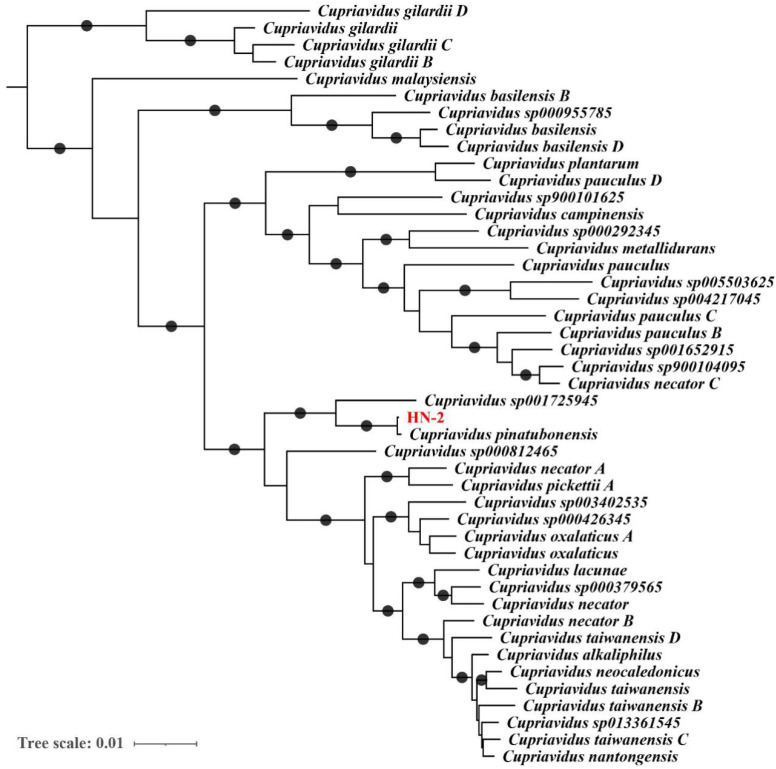
Phylogenetic relationship between *Cupriavidus* *pinatubonensis* HN-2 and other *Cupriavidus* species. *C*. *pinatubonensis* HN-2 was placed in a bacterial reference tree using GTDB-tk based on 120 preselected marker genes. The subtree that includes HN-2 and all other *Cupriavidus* species is shown, and HN-2 is highlighted in red. Branches with support values ≥ 95% are indicated by black dots.

Our previous 16S rRNA-based analysis classified strain HN-2 as a member of the *Cupriavidus* genus. To further determine the taxonomic assignment of strain HN-2, we analyzed its genome sequence using GTDB-Tk, which first placed strain HN2 into a prebuilt bacterial phylogeny based on a set of 120 proteins and then calculated average nucleotide identity (ANI) values between strain HN-2 and its close relatives in the tree. As a result, strain HN-2 was discovered to be the most closely related to *C. pinatubonensis* JMP134 (Figure 2), with an ANI value of 99.43%, much over the usually recognized prokaryotic species delimitation threshold of 95%. Therefore, we assigned strain HN-2 as a strain of *C. pinatubonensis* based on genome data analysis.

### 3.2. Identification and Cloning of the Gene Responsible for the DSF Degradation

DSF is an unsaturated fatty acid, and, therefore, the ability of the strain HN-2 to quench DSF-mediated QS probably depends on enzymes in the fatty acid degradation pathway. For identification of genes in charge of DSF degradation in strain HN-2, we selected eight genes for further investigation based on their functional annotations (domain structures of their protein products are shown in Figure S6); these genes encode four long-chain acyl-CoA synthetases (HN.2_GM000231, HN.2_GM003041, HN.2_GM005970, and HN.2_GM000743, which were annotated with the KO term K01897 and are also homologs of *rpfB*), three 3-hydroxyacyl-CoA dehydrogenases (HN.2_GM000404, HN.2_GM003236, and HN.2_GM004234, which were annotated with the KO term K07516), and one acyl-CoA dehydrogenase (HN.2_GM004237). To investigate whether these genes affected the degradation of DSF in strain HN-2, mutants with deletion of these genes were generated using strain HN-2 as the parental strain. Finally, the 0404 in-frame ∆0404 deletion mutant, the 3236 in-frame ∆3236 deletion mutant, the 4234 in-frame ∆4234 deletion mutant, the 4237 in-frame ∆4237 deletion mutant, the 0231 in-frame ∆0231 deletion mutant, the 3041 in-frame ∆3041 deletion mutant, the 5970 in-frame ∆5970 deletion mutant, and the 0743 in-frame ∆0743 deletion mutant were generated. After exploring their inhibitory effect on the pathogenicity of *Xcc*, GM004237 was initially identified as a DSF-degradation gene in strain HN-2 and named *fadT*. The other genes GM000231, GM003041, GM005970, GM000743, GM000404, GM003236, and GM004234 have no inhibitory effect on the pathogenicity of *Xcc*.

**Figure 3:**
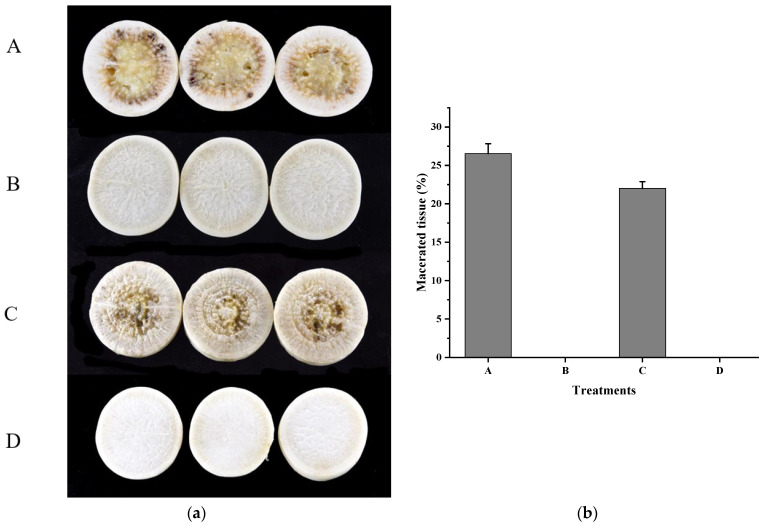
Biocontrol effect of *fadT* gene deletion on *Xcc* pathogenicity. (**a**) Radishes (*Raphanus sativus* L.) were inoculated with *Xcc* alone (**A**), *Xcc* + HN-2 wild-type (**B**), *Xcc* +HN-2∆*fadT* (**C**), and distilled water (**D**). The photograph was taken 2 days after inoculation. (**b**) Maceration area rate in each treatment.

The biocontrol experiment showed that strain HN-2 could well decrease DSF-mediated *Xcc* pathogenicity, whereas the *fadT* in-frame ∆*fadT* deletion mutant had no inhibitory effect on DSF-mediated *Xcc* pathogenicity. Biocontrol efficacy against black rot in Chinese radish of the control (CK), wild-type (strain HN-2), and ∆*fadT* is shown in Figure 3. It is worth pointing out that the wild-type strain HN-2 exhibited superior DSF degradation activity and completely degraded 2 mM of DSF within 24 h. In addition, ∆*fadY* could not degrade DSF, and ∆*fadT* (*fadT*) regained the ability to degrade DSF, which further indicated that *fadT* played a key role in the degradation of DSF. The comparative study of DSF-degrading activity between the control (CK), wild-type strain HN-2, ∆*fadT*, and ∆*fadT* (*fadT*) cultured in MSM medium for 24 h is shown in Figure S7. These results identified *fadT* as the key gene affecting wild HN-2 in the alleviation of bacterial virulence on host plants, and it was thus identified as an important gene responsible for DSF degradation.

### 3.3. Codon Optimization, Expression, and Purification of FadT

In order to obtain a higher yield and a pure DSF-degrading enzyme, the gene *fadT* was codon optimized. After the optimized gene co-*fadT* was obtained, it was synthesized and ligated into the vector pGEX-6p-1. In addition, pGEX-6p-1-co-*fadT* was obtained and then transferred into *E. coli* BL21. Finally, the engineered strain *E. coil* BL21/pGEX *fadT* was acquired, and it was abundantly expressed to obtain the crude enzyme extract, followed by affinity purification. As shown in Figure S8, the SDS-PAGE staining showed that GST-FadT had a molecular weight of about 93 kDa as predicted in silico. The band of GST-FadT was single, indicating the enzymes were of high purity. The purified FadT protein exhibited high enzymatic activity and outstanding stability over a broad pH (6.0–8.0) and temperature (25–45 °C) range with maximal activity at pH 7.0 and 35 °C. No cofactors were required for FadT enzyme activity. Moreover, the FadT enzyme showed a strong ability to degrade DSF (Figure S9).

### 3.4. Xcc Expressing fadT Loses Virulence in Planta

**Figure 4:**
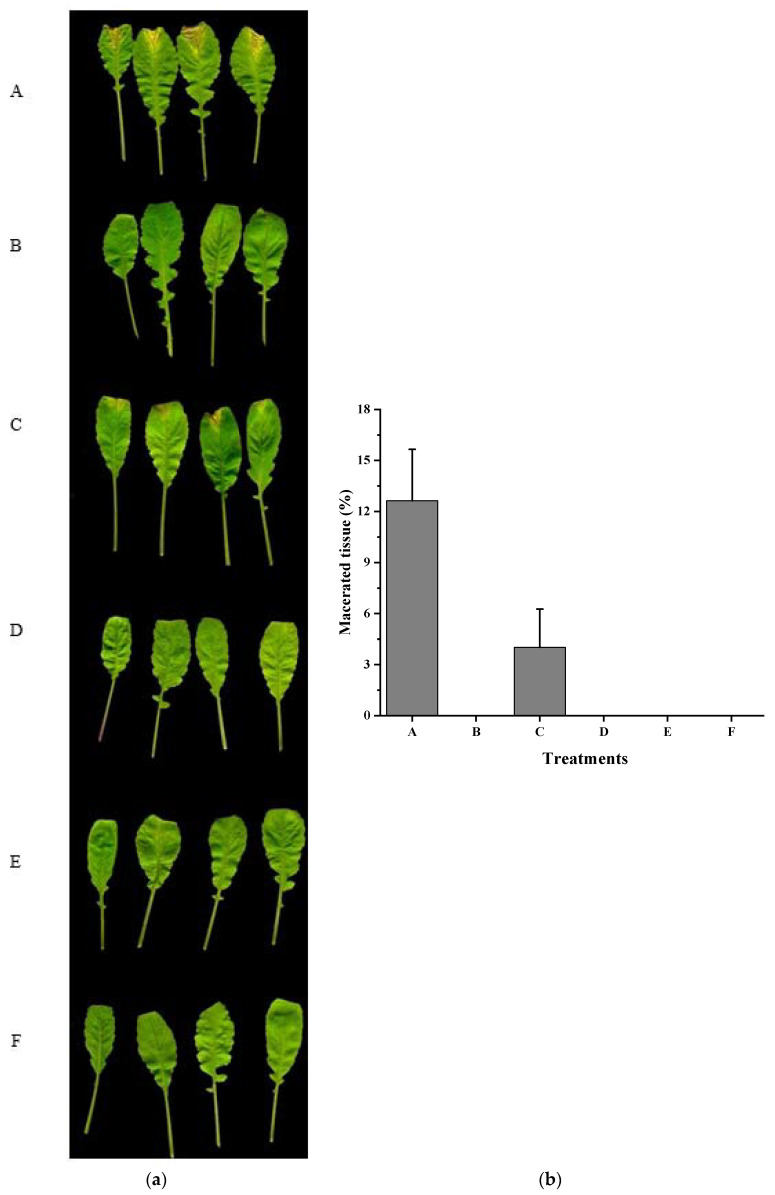
Effect of *fadT* gene expression on *Xcc* pathogenicity. (**a**) Radishes (*Raphanus sativus* L.) were inoculated with *Xcc* (**A**), *Xcc* + HN-2 (**B**), *Xcc* + HN-2∆*fadT* (**C**), *Xcc* + HN-2∆*fadT* (*fadT*) (**D**), *Xcc* (*fadT*) (**E**), and distilled water (**F**). (**b**) Maceration area rate in each treatment. Gene *fadT* was cloned into shutter vector pBBR1-MCS5 and mobilized into strain HN-2 or *Xcc*. Photograph was taken 8 days after inoculation.

**Figure 5:**
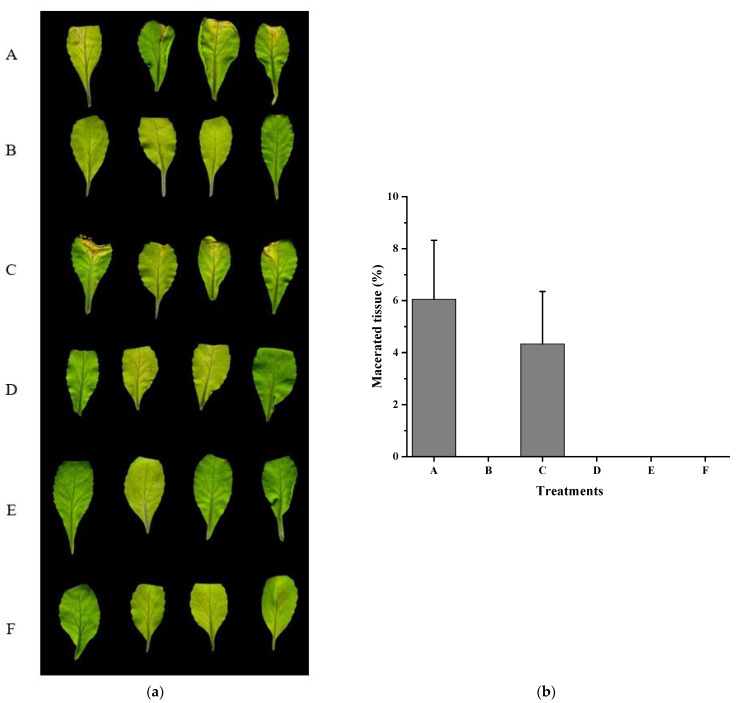
Effect of *fadT* gene expression on *Xcc* pathogenicity. (**a**) Chinese cabbages (*Brassica pekinensis* (Lour.) Rupr.) were inoculated with *Xcc* (**A**), *Xcc* + HN-2 (**B**), *Xcc* + HN-2∆*fadT* (**C**), *Xcc* + HN-2∆*fadT* (*fadT*) (**D**), *Xcc* (*fadT*) (**E**), and distilled water (**F**). (**b**) Maceration area rate in each treatment. *fadT* gene was cloned into shutter vector pBBR1-MCS5 and mobilized into strain HN-2 or *Xcc*. Photograph was taken 8 days after inoculation.

**Figure 6:**
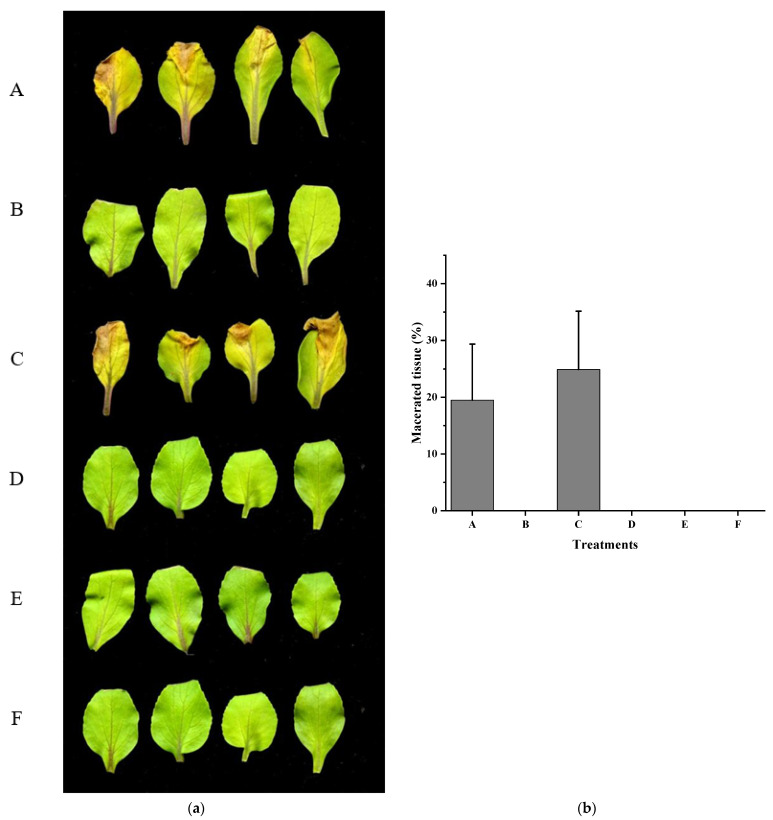
Effect of *fadT* gene expression on *Xcc* pathogenicity. (**a**) Pakchoi cabbages (*Brassica campestris* L. ssp. *chinensis* Makino) were inoculated with *Xcc* (**A**), *Xcc* + HN-2 wild-type (**B**), *Xcc* + HN-2∆*fadT* (**C**), *Xcc* + HN-2∆*fadT* (*fadT*) (**D**), *Xcc* (*fadT*) (**E**), and distilled water (**F**). (**b**) Maceration area rate in each treatment. *fadT* gene was cloned into shutter vector pBBR1-MCS5 and mobilized into strain HN-2 or *Xcc*. Photographs were taken 10 days after inoculation.

To explore the function of *fadT* gene in the biocontrol of *Xcc*, we performed plant inoculation experiments. Radish (*Raphanus sativus* L.), Chinese cabbage (*Brassica pekinensis* (Lour.) Rupr.), and pakchoi (*Brassica campestris* L. ssp. *chinensis* Makino), commonly used in the test of biocontrol of black rot disease, were inoculated with *Xcc* together with wild-type or *fadT*-deletion strain HN-2. Each tested plant inoculated with *Xcc* alone presented severe black rot symptoms (Figure 4, Figure 5 and Figure 6). In contrast, inoculation of a mixture of *Xcc* with wild-type HN-2 almost completely diminished the plant disease symptoms, suggesting wild-type HN-2 counteracts the virulent effects of *Xcc*. To demonstrate the role of *fadT* gene in strain HN-2, *Xcc* was further mixed with *fadT*-deletion HN-2 in the plant inoculation experiment. As shown, strain HN-2 without *fadT* gene could not impair the pathogenicity of *Xcc*, while complementation of *fadT* gene in the *fadT*-deletion HN-2 restored its biocontrol ability. These results further demonstrated the essential role of *fadT* gene in the interaction of strain HN-2. Further, on the test plants, the virulence of *Xcc* expressing HN-2-derived *fadT* gene was also reduced. Taken together, the expression of *fadT* gene could effectively attenuate the black rot disease caused by *Xcc* in the host plants.

## 4. Discussion

Understanding the genomic diversity of bacterial strain and the relevant bioactive products are of importance for disease control [59,60]. In this study, whole-genome sequencing (WGS) combined with genome annotation was applied to uncover the mechanism of DSF degradation in *Cupriavidus pinatubonensis* strain HN-2. The genome sequence of strain HN-2 discovered in this study will be useful in the development of DSF-degrading bacteria or enzymes in the future. Further studies require a focus on genes related to the pathway that metabolizes DSF.

The emergence of antibiotic-resistant bacteria causes more difficulties in the treatment of bacterial infectious diseases. As for the control of diseases caused by bacteria, the use of QQ is a promising avenue to replace traditional antibiotics. In principle, QQ quenches the QS molecules of targeted bacteria, resulting in the shutdown of the expression of QS-controlled virulence genes [61,62,63,64]. As a result, a variety of bacteria that degrade DSF have been found and characterized, including *Staphylococcus*, *Bacillus*, *Microbacterium*, *Paenibacillus*, *Cupriavidus*, *Acinetobacter*, and *Pseudomonas* [15,65,66,67,68,69]. However, the genome sequence and molecular mechanisms of DSF-degrading strains are not clear, which limits the applications of these QQ strains. Since the discovery of the first AHL lactase AiiA isolated from *Bacillus cereus* [66] and the AHL amidase isolated from *Variovorax paradoxus*, an increasing amount of AHL-degrading enzymes have been well identified [70,71,72,73,74].

DSF has multiple effects on various cellular processes of bacteria. It has been reported that DSF can block protease secretion from attack-phase cells of *Bdellovibrio bacteriovorus* 109 J completely at the concentration of 50 mM and negatively regulate the membrane integrity at a concentration of 200 mM [75]. Additionally, DSF inhibits biofilm formation with no impacts on the growth of *P. aeruginosa* under static and continuous conditions [76]. DSF also regulates intercellular public goods (such as extracellular polymeric substances (EPS) and amino acids) [76]. DSF-based QS systems are classified into three major classes, the first from *Xcc*, the second from *Burkholderia cenocepacia* and *Cronobacter turicensis*, and the third from *Pseudomonas aeruginosa* [77,78,79,80]. However, limited DSF-degrading bacteria and their corresponding enzymes were identified. This is the first research that we are aware of that describes a novel DSF-degrading enzyme in *C. pinatubonensis*.

Our findings show that expressing the *fadT* gene in *Xcc* significantly reduced black rot symptoms in the tested host plants, indicating FadT as a novel DSF-degrading enzyme with significant efficacy in reducing *Xcc* pathogenicity. Our study highlights the potential use of FadT in the biocontrol of *Xcc* and, as a starting point, indicates the significance of the application of FadT in a broader range of DSF-dependent bacterial pathogens.

## 5. Conclusions

In summary, our study first reports the genome sequence and identifies a novel DSF-degrading enzyme, FadT, in *C.* *pinatubonensis* HN-2. Plant experiments showed that *Xcc* carrying a functioning FadT markedly suppressed its virulence, resulting in the elimination of the symptoms of black rot in the assayed host plants. Our study demonstrates for the first time that FadT is a powerful tool for the biocontrol of black rot caused by *Xcc*. These novel findings reveal the biochemical characteristics of a highly efficient DSF-degrading bacterial isolate and provide useful enzymes/genes with great potential for the control of infectious diseases caused by DSF-dependent bacterial pathogens.

## Figures and Tables

**Table 1:** Strains and plasmids used in this study.

Strains or Plasmids	Relevant Genotype or Phenotype	Sources
HN-2 strains	Laboratory storage	This study
∆*fadT*	*fadT* deletion mutant of strain HN-2 with 4323-nt internal coding region deleted	This study
*Xcc* XC1	Pathogenic bacteria responsible for black rot	Lab collection
*Escherichia coli* strains		
DH5*α*	*spuE44* ∆*lacU169(ϕ80lacZ∆M15) hsdR17λpir recA1 endA1 gyrA96 thi-1 relA1*	Lab collection
BL21	F^−^ompThsdS (rB^−^mB^−^) dcm^+^ Tet ^r^ gal (DE3) *end*A	Lab collection
pRK2013	Tra^+^, Mob^−^, ColE1-replicon, Kan ^r^, Spe ^r^	Lab collection
Plasmids		
pBBR1-MCS5	Broad-host-range expression vector; Gm ^r^	Lab collection
pK18mobsacB	Broad-host-range gene knockout vector, sacB, Gm^r^	Lab collection
pBBR1-MCS5-*fadT*	pBBR1-MCS5 containing *fadT* under control of P_lac_	This study
pK18-*fadT*	pK18mobsacB containing flanking sequences of *fadT*	This study
pGEX-6p-1	GST fusion protein expression vector, Amp ^r^	Invitrogen
pGEX-*fadT*	pGEX-6p-1 containing *fadT*	This study

Note: Superscript “r” means “resistance”.

**Table 2:** Primers used in this study.

Primers	Sequence (5′–3′)	Purposes
De*fadT*upF	GAGCTCGGTACCCGGGGATCCGGCCCGGTGTTCGCGGTG	For amplification of the 5′-region of *fadT*
De*fadT*upR	ATAGCGGCGCGGGGGTGTCTCCATGCGGGGC	
De*fadT*dnF	AGACACCCCCGCGCCGCTATCCGTG	For amplification of the 3′-region of *fadT*
De*fadT*dnR	ACGACGGCCAGTGCCAAGCTTTCGATGCCACGTTGATGATG	
Ts*fadT*-F	CTTATGGATGTCCGGGGTCG	For identification of ∆*fadT*
Ts*fadT*-R	TTCATGACCTTGTCCCAGGC	
c*fadT*-F	GTCGACGGTATCGATAAGCTTAGTACGTCGTCGTGACGATGTG	For construction of pBBR1-MCS5-*fadT*
c*fadT*-R	CGCTCTAGAACTAGTGGATCCGGTTCCCTGATGTTCCTCGAT	
p*fadT*-F	TTCCAGGGGCCCCTGGGATCC ATGTCGCTGATCCTTTCCCG	For construction of pGEX-6P-1-*fadT*
p*fadT*-R	CTCGAGTCGACCCGGGAATTC TCAGAACCAGGCGTCCTGC	

## Supplementary Materials

The following are available online at https://www.mdpi.com/article/10.3390/ijms22189862/s1. Figure S1. Statistical diagram of gene distribution in the *Cupriavidus pinatubonensis* HN-2 gene island. Figure S2. Classification of *Cupriavidus pinatubonensis* HN-2 functional genes in the GO database. Figure S3. Classification of *Cupriavidus pinatubonensis* HN-2 functional genes in the COG database. Figure S4. KEGG metabolic pathway classification diagram of *Cupriavidus pinatubonensis* HN-2. Figure S5. CAZy functional classification and corresponding gene number statistics of HN-2. AA: oxidoreductase; CBM: carbohydrate-binding module; CE: carbohydrate esterases; GH: glycoside hydrolase; GT: glycosyltransferase; PL: polysaccharide lyase; it was not found in the KEGG metabolic pathway of *Cupriavidus pinatubonensis* HN-2. Figure S6. InterProScan predicted domain structures of proteins encoded by the eight genes selected for deletion assay. (A) Four long-chain acyl-CoA synthetases encoded by HN.2_GM000231, HN.2_GM003041, HN.2_GM005970, and HN.2_GM000743. Two characteristic domains were predicted, namely IPR000873 “AMP-dependent synthetase/ligase” and IPR025110 “AMP-binding enzyme, C-terminal domain”. (B) Three 3-hydroxyacyl-CoA dehydrogenases encoded by HN.2_GM000404, HN.2_GM003236, and HN.2_GM004234. Two characteristic domains were predicted, namely IPR006176 “3-hydroxyacyl-CoA dehydrogenase, NAD binding” and IPR006108 “3-hydroxyacyl-CoA dehydrogenase, C-terminal”. (C) The acyl-CoA dehydrogenase encoded by HN.2_GM004237, which was named fadT in our study. Four characteristic domains were predicted, namely IPR020953 “Acyl-CoA dehydrogenase, N-terminal, bacteria”, IPR006091 “Acyl-CoA oxidase/dehydrogenase, central domain”, IPR009075 “Acyl-CoA dehydrogenase/oxidase C-terminal”, and IPR025878 “Acetyl-CoA dehydrogenase-like C-terminal domain”. Figure S7. SDS-PAGE analysis of purified FadT. M: Marker; 7: Purified FadT. Figure S8. Effects of temperature and pH on FadT activity. (A) Determination of optimal temperature; (B) determination of optimal pH. Figure S9. Diffusible signal factor (DSF)-degrading activity comparison of mutants and wild-type strain HN-2. CK: control; strain HN-2: wild-type; ∆*fad**T*: *fad**T* deletion mutant; ∆*fad**T* (*fad**T*): the complement of the *fad**T* deletion mutant. Table S1. Genome characteristics of *Cupriavidus pinatubonensis* HN-2. Table S2. Prediction of noncoding RNA (ncRNA) in strain HN-2 genome.
