# Supplementary material for: Identification of FadT as a Novel Quorum Quenching Enzyme for the Degradation of Diffusible Signal Factor in Cupriavidus pinatubonensis Strain HN-2
Source: Int J Mol Sci. 2021 Sep 13;22(18):9862. doi: 10.3390/ijms22189862 (PMC8467058; doi:10.3390/ijms22189862)
Supplement: Supplementary file 1 [file ijms-22-09862-s001.zip › ijms-1316418-supplementary.pdf]

Supplemental Materials

Identification of FadT as a Novel Quorum Quenching Enzyme for the Degradation of Diffusible Signal Factor in *Cupriavidus pinatubonensis* Strain HN-2

Xudan Xu [1,2,†], Tian Ye [1,2,†], Wenping Zhang [1,2], Tian Zhou [1,2], Xiaofan Zhou [1,2,*], Weijun Dai [1,2,*] and Shaohua Chen [1,2,*]

[1] Integrative Microbiology Research Centre, State Key Laboratory for Conservation and Utilization of Subtropical Agro-Bioresources, Guangdong Province Key Laboratory of Microbial Signals and Disease Control, South China Agricultural University, Guangzhou 510642, China; xuxudan@stu.scau.edu.cn (X.X.); 20182047012@stu.scau.edu.cn (T.Y.); 20191047008@stu.scau.edu.cn (W.Z.); 20171021009@stu.scau.edu.cn (T.Z.)

[2] Guangdong Laboratory for Lingnan Modern Agriculture, Guangzhou 510642, China

* Correspondence: xiaofan_zhou@scau.edu.cn (X.Z.); daiweijun@scau.edu.cn (W.D.); shchen@scau.edu.cn (S.C.); Tel.: +86-20-8528-8229 (S.C.)

† Both authors contributed equally to this work.

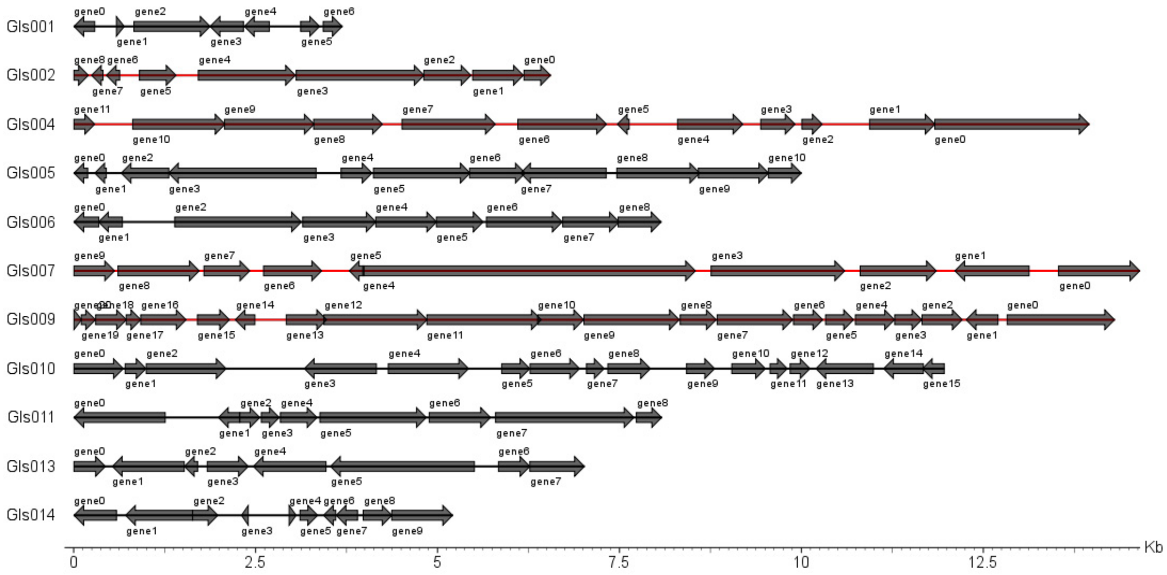

Figure S1. Statistical diagram of gene distribution in the *Cupriavidus pinatubonensis* HN-2 gene island.

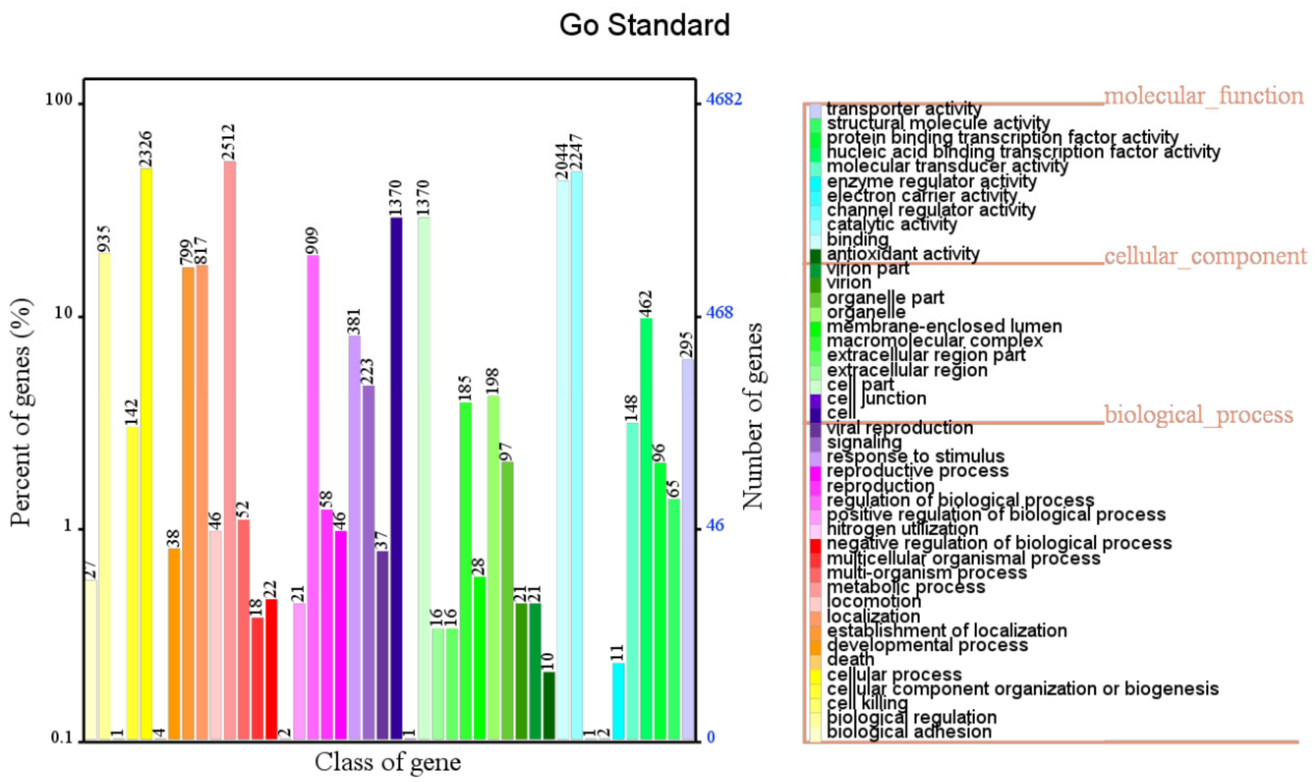

Figure S2. Classification of *Cupriavidus pinatubonensis* HN-2 functional genes in the GO database.

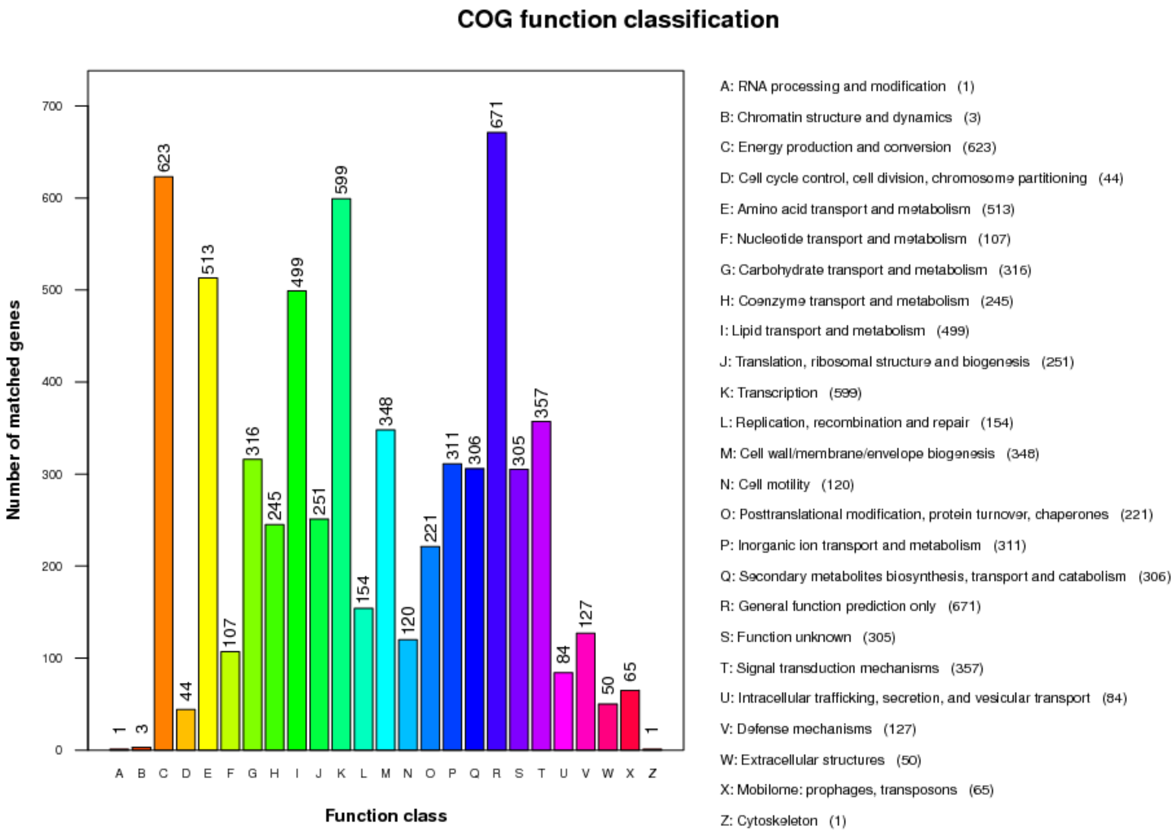

Figure S3. Classification of *Cupriavidus pinatubonensis* HN-2 functional genes in the COG database.

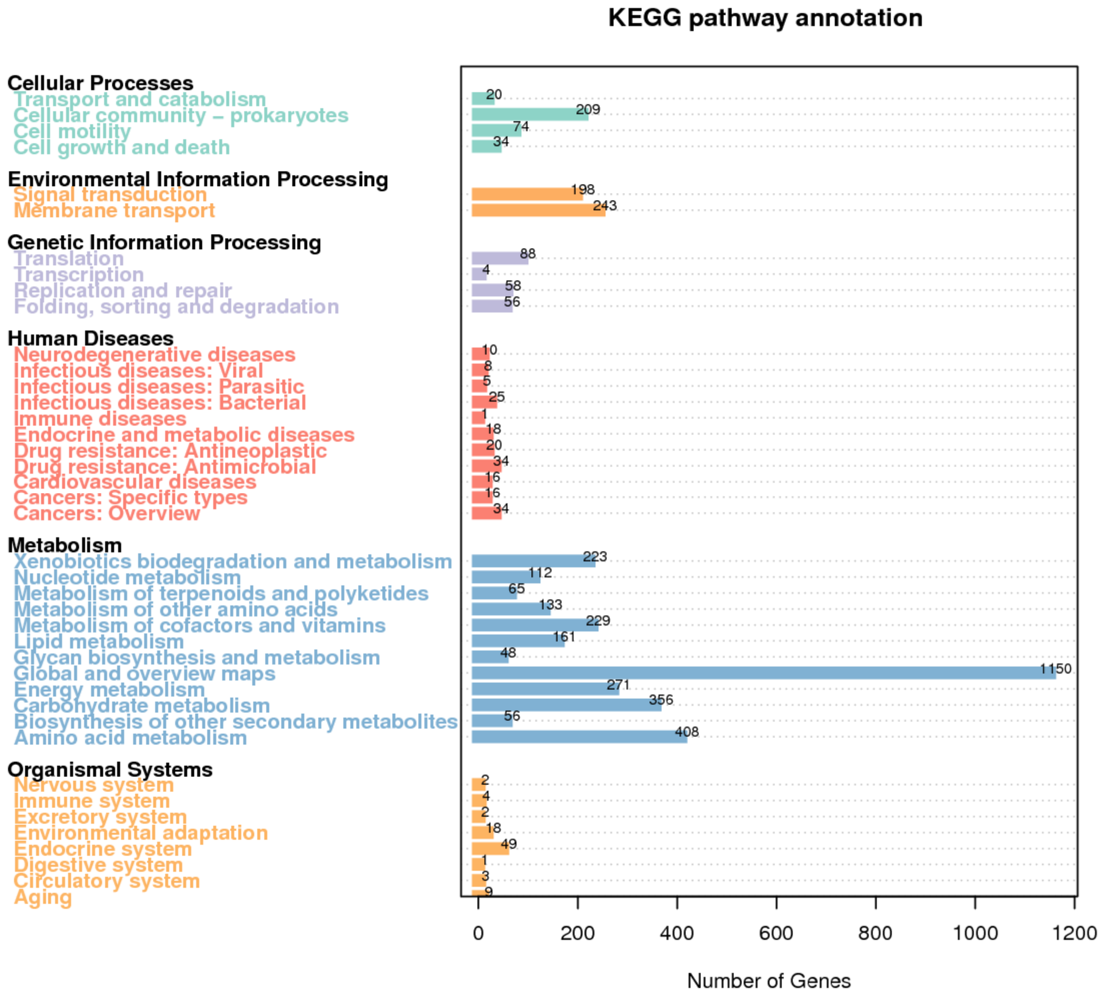

Figure S4. KEGG metabolic pathway classification diagram of *Cupriavidus pinatubonensis* HN-2.

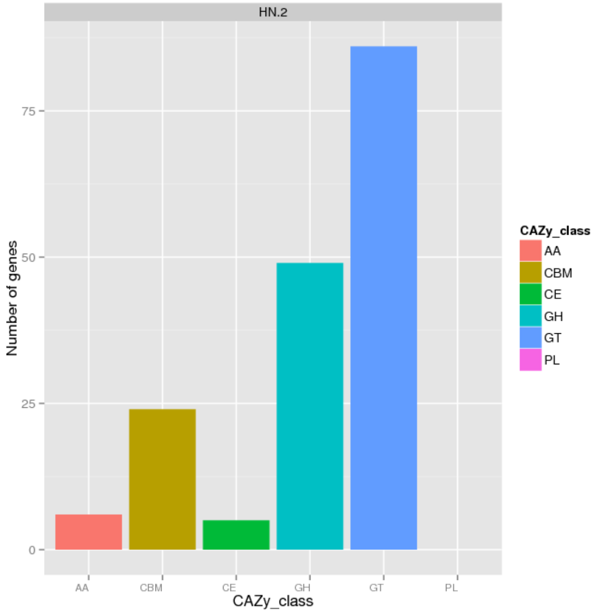

Figure S5. CAZy functional classification and corresponding gene number statistics of HN-2. AA: oxidoreductase; CBM: carbohydrate-binding module; CE: carbohydrate esterases; GH: glycoside hydrolase; GT: glycosyltransferase; PL: polysaccharide lyase; it was not found in the KEGG metabolic pathway of *Cupriavidus pinatubonensis* HN-2.

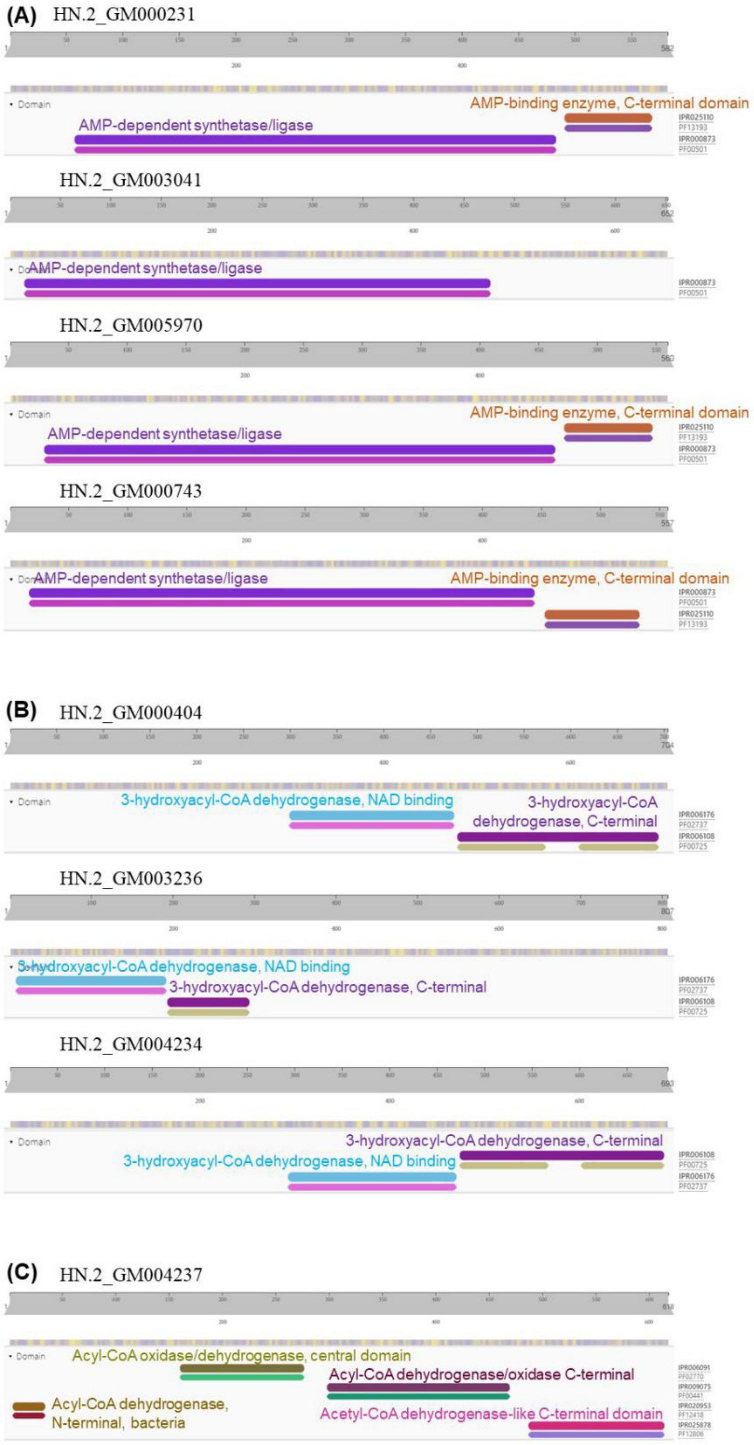

Figure S6. InterProScan predicted domain structures of proteins encoded by the eight genes selected for deletion assay. (A) Four long-chain acyl-CoA synthetases encoded by HN.2_GM000231, HN.2_GM003041, HN.2_GM005970, and HN.2_GM000743. Two characteristic domains were predicted, including IPR000873 "AMP-dependent synthetase/ligase" and IPR025110 "AMP-binding enzyme, C-terminal domain". (B)

Three 3-hydroxyacyl-CoA dehydrogenases encoded by HN.2_GM000404, HN.2_GM003236, and HN.2_GM004234. Two characteristic domains were predicted, including IPR006176 “3-hydroxyacyl-CoA dehydrogenase, NAD binding” and IPR006108 “3-hydroxyacyl-CoA dehydrogenase, C-terminal”. (C) The acyl-CoA dehydrogenase encoded by HN.2_GM004237, which was named fadT in our study. Four characteristic domains were predicted, including IPR020953 “Acyl-CoA dehydrogenase, N-terminal, bacteria”, IPR006091 “Acyl-CoA oxidase/dehydrogenase, central domain”, IPR009075 “Acyl-CoA dehydrogenase/oxidase C-terminal”, and IPR025878 “Acetyl-CoA dehydrogenase-like C-terminal domain”.

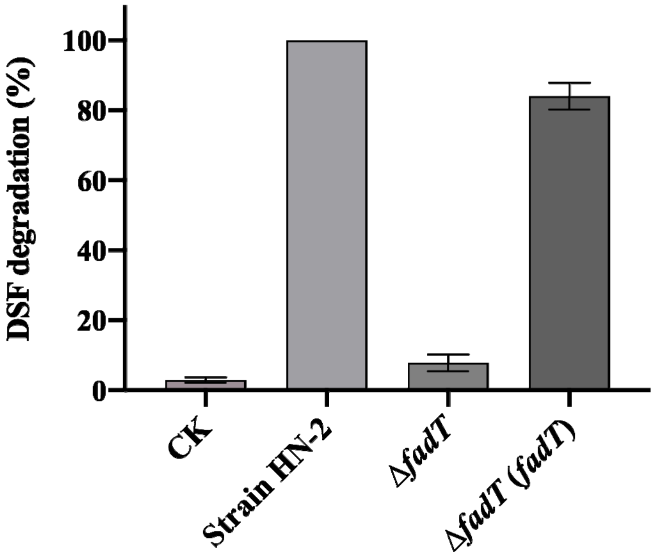

Figure S7. Diffusible signal factor (DSF)-degrading activity comparison of mutants and wild-type strain HN-2. CK: control; strain HN-2: wild-type; Δ*fadT*: *fadT* deletion mutant; Δ*fadT* (*fadT*): the complement of the *fadT* deletion mutant.

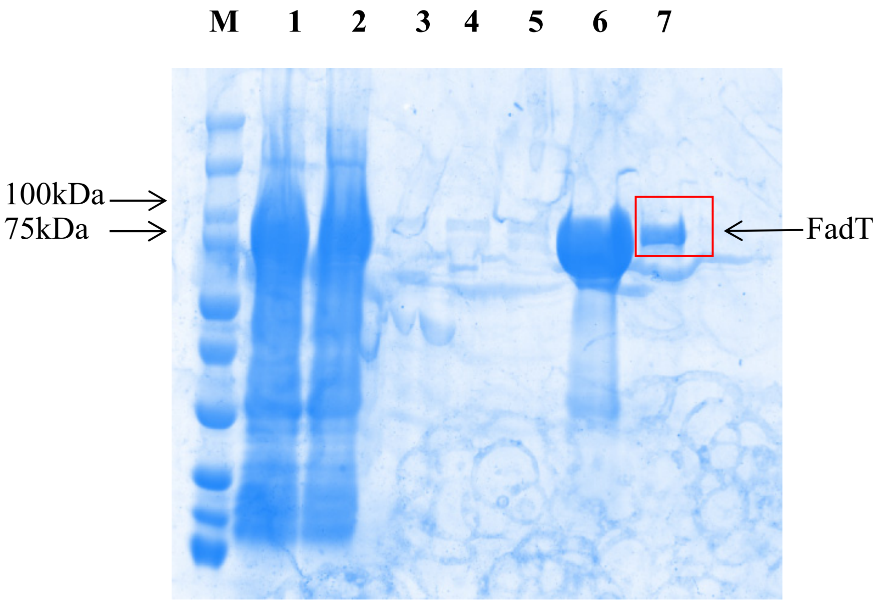

Figure S8. SDS-PAGE analysis of purified FadT. M: Marker; 7: Purified FadT.

A

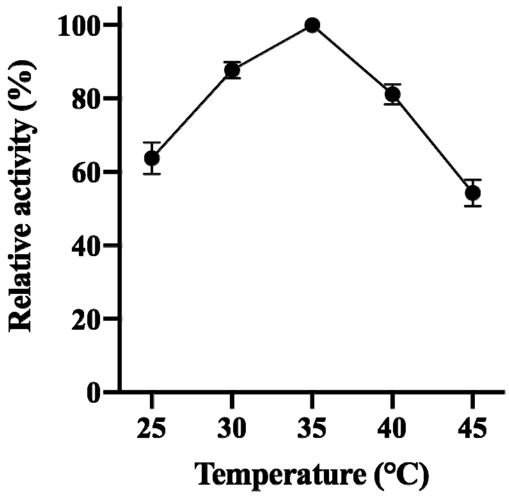

B

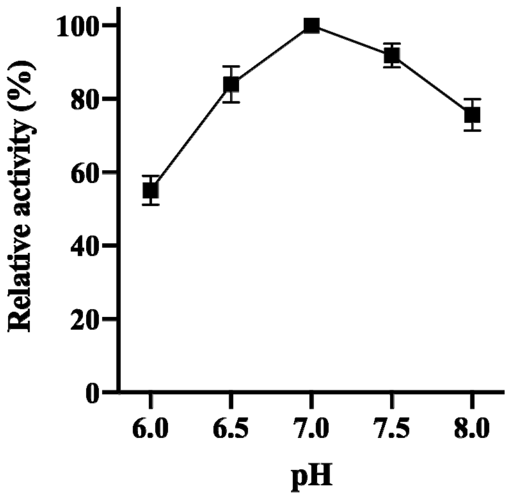

Figure S9. Effects of temperature and pH on FadT activity. (A) Determination of optimal temperature; (B) Determination of optimal pH.

Table S1. Genome characteristics of *Cupriavidus pinatubonensis* HN-2.

Genome Characteristics	
Total length (bp)	7,548,664
Number of protein-coding genes	7,101
Average length of protein-coding genes (bp)	923
% of Genome (protein-coding genes)	86.86%
rRNA genes	18
tRNA genes	65
sRNAs genes	1

Table S2. Prediction of non-coding RNA (ncRNA) in strain HN-2 genome.

**Type**		Quantity	Average length(bp)	Total length(bp)	Proportionof the whole genome(%)
tRNA		65	78	5,087	0.0674
rRNA_	5s rRNA	6	112	673	0.3587
	16s rRNA	6	1,522	9,130	
	23s rRNA	6	2,879	17,274	
sRNA		1	88	88	0.0012
